# Use of Robotic Devices in Upper Limb Rehabilitation for Adult Stroke Patients: Protocol for an Umbrella Review

**DOI:** 10.1002/hsr2.71684

**Published:** 2025-12-27

**Authors:** Vanesa Carrión‐Téllez, José‐Ángel Pastor‐Zaplana, Laura‐María Compañ‐Gabucio, Paula Peral‐Gómez, Nicolás‐Manuel García‐Aracil

**Affiliations:** ^1^ Departamento de Patología y Cirugía Universidad Miguel Hernández de Elche Alicante Spain; ^2^ Departamento de Patología y Cirugía, Being + Doing & Becoming Grupo de Investigación Ocupacional (B+D+b) Universidad Miguel Hernández de Elche Alicante Spain; ^3^ Universidad Miguel Hernández de Elche, Departamento de Paología y Cirugía. Unidad de Epidemiología de la Nutrición (EPINUT) Instituto de Investigación Sanitaria y Biomédica de Alicante (ISABIAL) Alicante Spain; ^4^ Departamento de Patología y Cirugía, Being + Doing & Becoming Grupo de Investigación Ocupacional (B+D+b). Instituto de Investigación Sanitaria y Biomédica de Alicante (ISABIAL) Universidad Miguel Hernández de Elche Alicante Spain; ^5^ Instituto de Bioingeniería Universidad Miguel Hernández de Elche Alicante Spain

**Keywords:** end‐effector devices, exoskeleton devices, research design, robotic devices, stroke, upper extremity rehabilitation

## Abstract

**Background and Aims:**

The sequelae of stroke have a profound impact on individuals' independence. Among these, motor limitations of the upper limb significantly impair activities of daily living. Currently, robotic therapies have emerged as an innovative therapeutic approach; however, the existing evidence is heterogeneous and fails to provide conclusive data regarding their effectiveness. Therefore, the objective of this umbrella review is to describe the effectiveness of robotic devices for upper limb rehabilitation in adults who have had a stroke. In addition, to identify the most commonly used robotic devices and examine the benefits of their use in combination with other therapeutic modalities.

**Methods:**

We will conduct an umbrella review with a systematic search in the following databases: PubMed (MEDLINE), Scopus, EMBASE, Web of Science, and the Cochrane Library. We will limit the search to the last 5 years in order to include the most recent literature. Search results will be independently screened by two authors in two phases: by title and abstract, and then by full text. We will include systematic reviews with and without meta‐analysis published in English, French, or Spanish aimed at exploring the use of robotic devices in upper limb motor rehabilitation for adults' post‐stroke.

**Results:**

Two authors will extract data into pre‐designed tables. We will include variables such as strength, joint range of motion, upper limb and hand motor function, spasticity, and dependence in activities of daily living. The AMSTAR‐2 scale will be used to assess the methodological quality of the included systematic reviews. Results will be presented narratively and in tables.

**Conclusion:**

This protocol will facilitate the development of an umbrella review that synthesizes current evidence on the effectiveness of robotic devices for upper limb rehabilitation after stroke, specifically identifying device classifications, therapeutic approaches, and clinical outcomes.

## 1 Introduction

Stroke is the second leading cause of death globally, accounting for nearly seven million deaths worldwide, and the third leading cause of disability [1]. It is defined as an acute, focal neurological deficit with no explanation other than a cerebrovascular cause [1]. Despite advances in prevention and treatment, stroke incidence has risen in several regions since 2015, underscoring the urgent need for effective strategies to mitigate this growing burden [2]. In 2021, there were 11.9 million new cases of stroke worldwide, with populations of lower socioeconomic status being disproportionately affected [3]. Factors such as population growth and aging contribute to the 70% increase in global stroke incidence and the 85% rise in its prevalence between 1990 and 2019 [1], further exacerbating the healthcare burden [2]. Additionally, Europe's elderly population is projected to grow by 35% in between 2017 and 2050 [4], which will intensify the challenges of stroke care. There is also growing concern about the rising incidence of stroke among young adults, particularly those aged 35 and older. This trend is especially pronounced among women aged 18–44 years, whose incidence of ischemic stroke has increased by 23% over the past decade [5]. Most individuals who survive a stroke encounter substantial difficulties, often involving complications that compromise their long‐term health and limit their ability to live independently [6]

Stroke can lead to a broad range of health consequences including cognitive impairment [7] such as memory and attention deficits, emotional effects such as depression and anxiety [8], social challenges [9] such as difficulties in engaging with others or returning to primary occupations like work or education, and functional limitations including dependency on others for daily living activities [10]. However, physical consequences are those that most limit functionality [11], especially motor impairment in the upper limb, which affects individuals' abilities to perform daily, work‐related, and social activities [12], and also contributing to emotional distress [8]. In this context, rehabilitation is a crucial aspect of the recovery process for people with stroke, as it is aimed at optimizing functionality, promoting independence, and improving quality of life in this population [13, 14].

In recent years, advanced robotic‐assisted rehabilitation has emerged as an innovative therapeutic intervention [15] because it can improve upper limb functionality [16] in patients with motor deficits secondary to stroke [17]. Robotic devices enhance upper limb performance [1] and enable more intensive practice for individuals with moderate to severe upper limb paresis following stroke [18]. In this regard, these devices allow for a more precise and adaptable intervention, showing more effectiveness in upper limb movement recovery than traditional rehabilitation [19]. Bhattacharjee et al. conducted a study combining robotic‐assisted therapy and daily living activities training and reported that this approach was more effective than combining daily living activities training with conventional therapy over 12 weeks of intervention [20]. Similarly, a crossover randomized clinical trial by Chen et al. found that robot‐assisted therapy was as effective as task‐oriented training in terms of improving upper limb functional performance within the activity domain [21]. In line with these experimental findings, systematic reviews (SRs) by Yang et al. [22] and Su et al. [16], concluded that robotic device rehabilitation is superior to conventional training and can significantly improve both motor function of the upper limb and daily living activities in persons with stroke. However, Carrillo et al. [23], in their SR, reported insufficient evidence to support that the use of robotic devices accelerates upper limb recovery when used alongside conventional therapy. Additionally, a SR by De Laco et al. [24] found that the modest effects observed with robotic devices in improving upper limb motor disability did not translate into clinically meaningful improvements in upper limb capacity.

While numerous SRs and other review studies on this topic are available the low quality of many of these may undermine the reliability of the results [25]. It is crucial to highlight the heterogeneity of studies published to date [26], in terms of design, methodological quality, population characteristics, and clinical variables assessed. This variability complicates the extraction of robust conclusions and the generalization of findings [25]. In light of this, there is a pressing need for a standardized intervention protocol that would provide clear, updated, and unified guidelines. Such a protocol would facilitate data analysis across different studies, promote the development of more effective, evidence‐based rehabilitation strategies, and support healthcare professionals involved in stroke rehabilitation.

In this context, this umbrella review (UR) seeks to answer the following research question: Which is the effectiveness of robotic devices in upper limb rehabilitation for adult stroke patients, and how do different types of robotic devices affect motor function, strength, joint range of motion, spasticity, and dependence in activities of daily living? Consequently, our main objective is to describe the effectiveness of robotic devices for upper limb rehabilitation in adults who have had a stroke as well as to identify the most commonly used robotic devices and examine the benefits of their use in combination with other therapeutic modalities. The future results of this UR are expected to contribute to a better understanding of current trends in robotic upper limb rehabilitation after stroke. Furthermore, the findings may facilitate the identification of key elements such as patient profiles, device characteristics, and intervention parameters that could inform the development of standardized and evidence‐based clinical protocols. While not intended to establish clinical guidelines, the future results may serve as a preliminary framework to support the design of future research and the refinement of rehabilitation strategies.

## 2 Materials and Methods

### 2.1 Study Design

This protocol outlines an UR, registered in the International Prospective Register of Systematic Reviews (PROSPERO) following the recommendations [27] under the number CRD42024547375 and has been developed following the guidelines of the Preferred Reporting Items for Systematic Review and Meta‐Analysis Protocols (PRISMA‐P) [28]. This UR will adhere to the recommendations established by the reporting items for systematic reviews and meta‐analyses (PRISMA) [29], thereby ensuring both scientific rigor and comprehensive content. We selected an UR because there is a growing number of SRs with inconclusive findings on our study topic which suggests the need for an updated synthesis of the available evidence [30]. This research does not require ethical approval, as it involves the analysis of previously published secondary data.

### 2.2 Search Strategy

In line with methodologies used in previous studies [31], a comprehensive literature search will be conducted across four multidisciplinary databases and one specialized database. Based on this strategy, an optimal combination of databases to cover the most published evidence hat is composed of PubMed (MEDLINE), Scopus, EMBASE, and Web of Science, complemented by the search in a specific database in relation to our study aim, in this case, Cochrane Library.

We will use the same search equation in each consulted database, which will be structured according to the PICO format: adult and stroke (Population), robot* and exoskeleton (Intervention), “upper extremity” (Outcome). All search terms will be combined using the Boolean operators AND and OR. The detailed search strategy is presented in Table 1.

**Table 1:** Search strategy.

Population	#1	Adult AND stroke
Intervention	#2	Robot* OR exoskeleton
Comparaison	NA	NA
Outcome	#3	“Upper extremity”
Combined query	#1 AND #2 AND #3	

#### 2.2.1 Eligibility Criteria

SRs must meet the following criteria in order to be included in this UR:

- Published in Spanish, English, or French.
- Designed as SRs, with or without meta‐analysis.
- The study population consists of adults ( > 18 years) with stroke in any phase (acute, subacute, or chronic).
- Focus on interventions mediated by robotic devices, exoskeletons or end‐effector robots aimed at upper limb rehabilitation, specifically targeting strength, joint range of motion, upper extremity and hand motor function, spasticity, and dependency in activities of daily living.
- Available in full‐text.

We will apply the exclusion criterion “full text not available” when an article is not open access, and we cannot obtain the complete version through our university library or by contacting the corresponding author of the article. If no response is received, this will be documented and reported in the final review. In addition, a 5‐year publication filter will be set, as the current scientific context in health sciences suggests that references should be recent to ensure information relevance [32]. This is particularly important in a field such as robotic rehabilitation, where technological advances occur rapidly [33].

SRs that do not meet the indicated inclusion criteria will be excluded.

#### 2.2.2 Study Selection and Screening

The study selection process will be conducted using Microsoft Excel. Once searches across the five databases are completed, all titles will be consolidated into a single spreadsheet, where a template with inclusion and exclusion criteria will be created to facilitate screening. The first column will contain the titles, the second will indicate inclusion status (yes/no), and the third column onwards will contain the exclusion criteria to be checked. Each time an article is excluded, the most relevant criterion will be selected. This template will be created before conducting the literature search to prevent data manipulation and ensure transparency among researchers during the screening process.

Two researchers (*anonymized*) will independently conduct the selection process. Any discrepancies that arise during this process regarding the inclusion or exclusion of an article will be addressed by a third researcher (*anonymized*). The study selection will be carried out meticulously by removing duplicate articles. Duplicate articles will be removed from the Excel database containing all the titles found in the five previously mentioned databases. To avoid potential errors when using the available Microsoft Excel functionalities for this task, the process will be carried out as follows: first, we will arrange the titles in alphabetical order, which will place titles starting with symbols or special characters, such as brackets or parentheses, at the beginning of the Excel sheet. Second, we will manually remove these special symbols. Third, we will reorder the titles alphabetically again to achieve a true alphabetical order, and finally, we will proceed with the elimination of duplicates.

After removing the duplicate titles, we will apply the inclusion criteria to the remaining articles through a two‐step selection process. In the first phase, both researchers (*anonymized*) will review titles and abstracts, and in the second phase, they will review full texts, using separate Excel sheets for each phase. Only articles that clearly do not meet the inclusion criteria will be excluded. In addition, we will complement our screening process by using Covidence, a software for systematic reviews, which allows for better documentation, transparency, and reproducibility of the study selection process [34].

#### 2.2.3 Data Extraction

**Figure 1:**
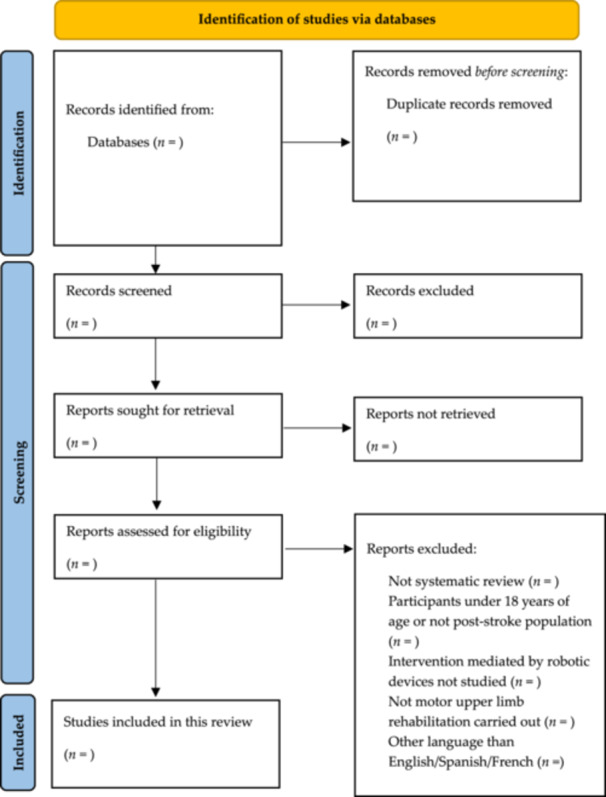
PRISMA flowchart for study selection.

The study selection and screening process will be presented using a PRISMA flow diagram [35] (Figure 1). A synthesis of the included SRs will be provided, both narratively and through tables and figures, to facilitate visualization and understanding of the findings.

Prior to data extraction, three tables will be designed to collect information from the articles, with predefined elements to ensure a more objective data synthesis and prevent data manipulation. The results will be reported following the recommendations of the Cochrane Handbook version 6.5 [36]. In accordance with the manual's guidelines, the results will be displayed in three tables: the first will outline the general characteristics of the included studies, the second will focus on characteristics specifically relevant to our research question, and the third will present information related to the methodological quality of the included systematic reviews. The first table, *“General Characteristics of Included Reviews”* will include elements such as authors' names, year of publication, number of included studies, total number of stroke patients, intervention/comparison, design of included studies, quality assessment tool used, whether a meta‐analysis was conducted and outcomes. The second table, *“Summary of Results,”* will contain data such as authors' names, intervention description used, intervention duration and results. Finally, a third table summarizing the quality assessment of each included SR will be presented. Two researchers (anonymized) will be responsible for extracting the data into the tables. All the researchers will collaborate in the narrative synthesis of the results and will be structured into categories such as motor function, spasticity, strength, joint range of motion, independence in activities of daily living, and stroke phase (acute, subacute, or chronic).

#### 2.2.4 Quality Assessment of SRs

The methodological quality of the included SRs will be evaluated using the critical appraisal tool, AMSTAR‐2 tool [37], which provides a rigorous framework for assessing the quality of SRs and the validity of their conclusions. This tool consists of 16 items for assessing different domains related to the methodological rigor of SRs. Eleven of these domains (items 1, 3, 5, 6, 10, 11, 12, 13, 14, 15, and 16) are rated dichotomously (yes/no), depending on whether the SRs meet the criteria for standard methods and reporting. The remaining five domains (items 2, 4, 7, 8, and 9) are rated with three options (yes/no/partially yes) [38].

Among the 16 items in AMSTAR 2, the following seven are considered “critical” as they critically affect the validity of SRs and its conclusions [38]:

◦ Registered prior protocol to outline the review methods and justify any deviations from the established protocol (item 2)
◦ Use of a comprehensive literature search strategy (item 4)
◦ Providing a clear list of excluded primary studies and rationalizing their exclusion (item 7)
◦ Assessing the risk of bias in primary studies (item 9)
◦ Adopting suitable and validated methodologies in the SR (item 11)
◦ Accounting for the risk of bias in primary studies when interpreting the results of the SR (item 13)
◦ Assessing and addressing publication bias, and discussing its potential influence on the SR results (item 15)

The included SRs will be independently evaluated by three reviewers (anonymized) using the AMSTAR‐2 tool. This instrument assesses the methodological quality of SRs across 16 domains, including several critical items such as protocol registration, adequacy of the search strategy, risk of bias assessment in primary studies, and consideration of publication bias. Based on the presence or absence of these methodological elements, each SR will be categorized into levels of overall confidence in quality (critically low, low, moderate, or high), following the guidance established by the developers of AMSTAR‐2 [37]. Any discrepancies between reviewers will be resolved through discussion with a fourth reviewer (anonymized).

#### 2.2.5 Overlap of Primary Studies

We will calculate the total overlap (RCTs included in the SRs) as well as the overlap by stroke phase, using the formula proposed by Pieper et al. [39]. Overlap will be reported as a percentage and as the Corrected Covered Area (CCA), which will be interpreted as follows: 0%–5% = slight overlap, 6%–10% = moderate overlap, 11%–15% = high overlap, and > 15% = very high overlap.

## 3 Limitations and Strengths

Our UR may have certain limitations that could influence our results. Although a systematic peer review will be conducted to ensure scientific rigor, the possible lack of reported information in the included SR, publication bias limiting the publication of null results, and selection bias are common limitations in most reviews. We will exclude SR not written in English, French, or Spanish, which may lead to the loss of potentially important information, thereby increasing selection bias. However, most relevant publications on this topic are written in English. Selection bias can also be increased by the fact that we will exclude any SR for which the full text is unavailable, although we will attempt to obtain it through our university's library services or by contacting the corresponding author. In this sense, the search will be conducted in only five databases, meaning that some important articles from other databases may be excluded. However, these five databases are among the most comprehensive and recommended for literature searches in reviews [31]. Although the 5‐year time frame enhances the relevance and currency of the evidence included, it may also increase the risk of selection bias by excluding high‐quality SRs published slightly outside this period; therefore, it should be considered with caution [40]. Finally, we may encounter SRs with highly heterogeneous interventions, which could make it challenging to compare and unify the findings in the final analysis.

This UR will also present several strengths. The quality of each SR included will be assessed by three researchers using the critical appraisal tool, AMSTAR‐2, and we will include a table specifying the methodological quality of each, ensuring transparency of the results obtained. In addition, this review will provide a detailed description of the most commonly used robotic devices in upper limb rehabilitation for stroke patients, as well as their effectiveness and potential combinations with other therapeutic options. This will provide professionals in the field with the opportunity to access relevant information for their clinical practice. Our findings may serve as a foundation for developing a standardized intervention protocol that unifies patient inclusion and exclusion criteria, robotic device characteristics, and treatment design and parameters. This will facilitate study comparisons, strengthen the available evidence, and promote evidence‐based practice.

## 4 Conclusions

This UR seeks to provide a clear and up‐to‐date synthesis of the evidence regarding the use of robotic devices in upper limb rehabilitation for persons who have suffered a stroke. Our goal is to propose a standardized intervention protocol using robotic devices in stroke rehabilitation, based on the findings of this review. This protocol will include guidelines on key aspects such as the choice of robotic devices, as well as the recommended frequency and duration of therapy sessions to optimize outcomes. The implementation of this protocol is expected to facilitate comparisons between future studies and promote a more effective, consistent clinical approach, providing a clear framework for healthcare professionals involved in neurological rehabilitation. Likewise, the findings may guide clinicians in implementing more effective rehabilitation strategies, thereby improving the quality of life of affected patients.

## Author Contributions

**Vanesa Carrión‐Téllez:** conceptualization, methodology, writing – original draft, data curation, supervision, writing – review and editing. **José‐Ángel Pastor‐Zaplana:** data curation, writing – review and editing. **Laura‐María Compañ‐Gabucio:** methodology, data curation, supervision, writing – review and editing. **Paula Peral‐Gómez:** conceptualization, methodology, supervision, writing – review and editing. **Nicolás‐Manuel García‐Aracil:** writing – review and editing, conceptualization.

## Disclosure

The lead author Vanesa Carrión‐Téllez affirms that this manuscript is an honest, accurate, and transparent account of the study being reported; that no important aspects of the study have been omitted; and that any discrepancies from the study as planned (and, if relevant, registered) have been explained. All authors have read and approved the final version of the manuscript. Vanesa Carrión‐Téllez had full access to all of the data in this study and takes complete responsibility for the integrity of the data and the accuracy of the data analysis.

## Conflicts of Interest

The authors declare no conflicts of interest.

## Data Availability

Data sharing not applicable to this article as no datasets were generated or analysed during the current study.
